# Book Reviews

**Published:** 1916-02

**Authors:** 


### BOOK REVIEWS

A TREATISE ON THE PRINCIPLES AND PRACTICE OF MEDICINE, by Arthur R. Edwards, M. D., Professor of the Principles and Practice of Medicine and Clinical Medicine and Dean of the Northwestern University Medical School, Chicago. New (third) edition, thoroughly revised. Octavo, 1022 pages, with 80 engravings and 23 full-page plates in colors and monochrome. Cloth, $600, net. Lea & Febiger, Philadelphia and New York, 1916.

The third edition of this valuable book has been demanded and the answer has been given in bringing the former editions up to date and yet keeping the same method that has made them acceptable to a discriminating set of men. Indeed there could be no radical change of method since it is the result of the careful training the author has given to himself in teaching medicine to students of all ages and conditions. There has been a resultant of forces of preparation, hard work and clear observation of the problems of life as they come to the physician which shows in the book and in the method of teaching it follows.

There is simplicity and directness of discussion of the diseases and clearness as to ideas of treatment that make this an ideal text book for the undergraduate and the best of references for the busy practitioner· Recent advances in therapeusis have been marked and we here have X-ray plates telling us the story of the stomach that even surgery did not disclose and indicating what must be done. Drugs and remedies are not only mentioned but care is taken to tell of their use so that one does not have to hunt up another book after learning that the disease under consideration needs certain things. The superior indifference of some of the older authors to this aspect of practise has been banished and in its place the reader will find information in detail.

There is necessarily condensation in the structure of the book because of the immense field it has to cover, but this never done at a sacrifice of information. One learns early that he has to read thoughtfully and that there is not a sentence of padding to be skipped.

The new remedies and methods, the great modern tests and symptoms and the special laboratory methods of diagnosis are all comprehensively handled and the discussion of them frankly tells one what a teacher believes he wants to know.

---

### PELLAGRA—SECOND EDITION

PELLAGRA, By George M. Niles, M. D., Gastro-enterologist to the Georgia Baptist Hospital, Wesley Memorial Hospital and Atlanta Hospital, Atlanta, Georgia. Octavo of 261 pages, illustrated. Philadelphia and London: W. B. Saunders Company, 1916. Cloth, $3.00 net.

In the second edition of this book given to the public upon one of the most important matters it can possibly have for its consideration, the author has faithfully brought all the information we have for our enlightenment. His own experience has been extensive and his observations are keen. Like all of us, he is hampered by the fact that no one knows exactly what

Pellagra is. The symptom complex of the disease is so very complicated and the pictures are so variable that accuracy and decision at this time are impossible.

But we are brought to look at the disease clearly as we would see it clinically and then there are given to us all the historic ideas concerning it and the soul of truth in each of these is brought out. We can not avoid noting what seems to us a curious attitude of thought on the part of all investigators in regard to the idea that the semi-drying oils may have a causative influence in Pellagro. Of course it is not the function of this author to elaborate a theory which as he says is not proved and no criticism is implied for his leaving it there, but when there remains a doubt as to the disease, everything is worthy of investigation that seems to have the least bearing upon the question.

The book has many quotations from well known students and these are brought together and held there by the author's studies and a style of composition which entices one to read every word he has to say. One feels that he knows what has been done to-date and that he has equipment for the care and prevention of the disease.

The latest thing the Marine Hospital issued as to its experiments in Mississippi penitentiary came too late for incorporation in this book, but it is noteworthy that in this as well as in the first edition, the author said that spoiled carbohydrates had grave influence upon the health and that they were potent in causing Pellagra.

---

### CANCER OF THE STOMACH

CANCER OF THE STOMACH, a Clinical Study of 921 Operatively and Pathologically Demonstrated Cases, by Frank Smithies, M. D., Gastro-enterologist to Augustana Hospital, Chicago. With a Chapter on the Surgical Treatment of Gastric Cancer, by Albert J. Ochsner, M. D., Professor of Clinical Surgery in the University of Illinois.

Octavo of 522 pages with 106 illustrations. Philadelphia and London: W. B. Saunders Company, 1916. Cloth, $5.50 net; Half Morocco, $7.00 net.

The book is founded upon the experience of "921 operatively and pathologically demonstrated instances of gastric cancer." It is the first monograph on the subject that has attained the dignity of book form in ten years and its purpose is to bring together all that has been gained during that time and to consider what place we occupy concerning this highly fatal disease.

In doing these things the material of large clinics, notably the Mayo's and the University Hospital at Ann Harbor have been used and added to these are the cases from the writer's more private work.

The whole subject of Gastric Cancer has been deeply studied and the judgments submitted impress everyone as consevative and careful. Several old ideas are set aside as useless and possibly superstitious. They are well out of the way. The new ideas are put forth with such support as they seem to merit. For instance it is noted that people using uncooked greens and vegetables have considerable increase in cancer and these comprise two interesting groups: The country people who can not afford anything else and the prosperous city people who can afford to buy these luxuries at a distance from their production. This will give a hint of the extent of the investigations that have been made.

The structure of the book is systematic and its chapters are conveniently divided so that one reads with comfort and the feeling that he is getting the story well in hand. The illustrations are numerous and the Roentgen plates are excellent and clear. These last alone reveal enough to make the book invaluable to the man who realizes that he must do more than guess at the presence of malignancy and that he can not afford to wait until it announces itself beyond a peradventure of a doubt. Indeed, the soul of the book strives to make and to teach an early diagnosis so that remedial agents shall be of avail. These are frankly and decisively surgical and there is no bargaining with other methods. The surgical methods and the various operations are handled in a special part of the book by Ochsner out right along with them there come suggestions as to non-surgical treatment when it is inevitable.

The relations of gastric cancer to other diseases either concomitant or otherwise associated are carefully presented and the modern ideas as to seriology, vaccination radiology and the like are weighed.

Every man doing gastric surgery should read it and the internists and family doctors who first see the cases of gastric derangement should become familiar with its teachings and guiding principles.

---

### SURGICAL OPERATIONS WITH LOCAL ANESTHESIA

Second Edition by Arthur E. Hertzler, A. M., M. D., Ph. D., F. A. C. S. Surgeon to the Halsted Hospital, Kansas, Swedish Hospital, Kansas City, Mo., General Hospital, Kansas City, Mo. 327 Pages; 173 Illustrations; Cloth Bound; Price $3.00. Surgery Publishing Company, New York.

The demand for the second edition of this work shows that the importance of local anaesthesia has had increasing recognition among surgeons. The study here presented tells of major as well as minor work and it startles one to think that abdominal sections are successfully accomplished without the shock one might expect in a conscious patient. The anaesthetics, themselves have been carefully investigated and the experience of the author is given as his authority for many unusual things. He has not been radical, however, for he has correlated the experience of other investigators to his own and he has not advised bizarre things or advocated sensational methods.

In the use of cocaine on the nasal mucosae, he does one thing we shall always condemn and that is advising the use of the spray. We can conceive of no condition where this would be advisable and certainly there is none where it is necessary. There is too much risk attending it to let it pass unnoticed.

The book is convenient in size and it should be a great assistance to men hesitating upon this important subject.

It is copiously and clearly illustrated so that one can begin his work along this line with confidence that he is doing the right thing in making his injections. The whole idea of "blocking" is nicely worked out and made plain and then the operator is shown that one great advantage in surgery is a corollery of this method. Gentleness is impressed upon him as it is far less likely to be when general anaesthesia is used. The benefit to the patient goes without saying.

---

PAINLESS CHILDBIRTH EUTOCIA AND NITROUS OXID-OXYGEN ANALGESIA, by Dr. Carl Henry Davis, Associate in Obstetrics and Gynecology, Rush Medical College in affiliation with the University of Chicago, Assistant Attending Obstetrician and Gynecologist to the Presbyterian Hospital, Chicago. Cloth, 134 Pages, $1.00 net. Chicago, Forbes & Company, 1916.

During the last two years there has been much said in the public prints as to painless childbirth and the profession has had to listen to the demands of the press that the Twilight sleep be granted to all women for the surcease of their labor pains.

Magazine writers and clever editors of the dailies have attempted to place the doctors in dilemas from which there appeared no escape save by the avenues they assumed to point out. They asked questions which were unanswerable to them because they were not well enough informed to comprehend the answers. Then they said the doctors were attempting to hide behind classic mystery of practise or to cover unwillingness to learn by pedantry. The lay demand was such as to embarass many a family doctor when he knew that the method asked for was fraught with danger.

In the book before us there appears to be a good plan for the relief of the major pains that mothers have to suffer. The book is written for the profession and will not be likely to attract other attention. It tells of the use of nitrous oxid-oxygen in obstetrics and details a large amount of experience on the part of the author. The dentists have been using analgesia successfully for a long time and the apparatus they have had constructed enables one to use the gas safely until he can become proficient with it and take advantage of the suggestions the book makes in this regard.

The book also takes up the important matter of puerperal sepsis and urges for obstetrics generally the efforts of every thoughtful man toward a reduction of this condition.

